# Review of Energy Management Methods for Fuel Cell Vehicles: From the Perspective of Driving Cycle Information

**DOI:** 10.3390/s23208571

**Published:** 2023-10-19

**Authors:** Wei Wang, Zhuo Hao, Fufan Qu, Wenbo Li, Liguang Wu, Xin Li, Pengyu Wang, Yangyang Ma

**Affiliations:** 1 CATARC Automotive Test Center (Tianjin) Co., Ltd., Tianjin 300300, China; wangwei2011@catarc.ac.cn (W.W.);; 2 CATARC NEV Test Center (Tianjin) Co., Ltd., Tianjin 300300, China; haozhuo@catarc.ac.cn; 3 College of Automotive Engineering, Jilin University, Changchun 130022, China; 4 School of Automotive Studies, Tongji University, Shanghai 201804, China

**Keywords:** energy management methods, fuel cell vehicles, driving cycle information

## Abstract

Energy management methods (EMMs) utilizing sensing, communication, and networking technologies appear to be one of the most promising directions for energy saving and environmental protection of fuel cell vehicles (FCVs). In real-world driving situations, EMMs based on driving cycle information are critical for FCVs and have been extensively studied. The collection and processing of driving cycle information is a fundamental and critical work that cannot be separated from sensors, global positioning system (GPS), vehicle-to-vehicle (V2V), vehicle-to-everything (V2X), intelligent transportation system (ITS) and some processing algorithms. However, no reviews have comprehensively summarized the EMMs for FCVs from the perspective of driving cycle information. Motivated by the literature gap, this paper provides a state-of-the-art understanding of EMMs for FCVs from the perspective of driving cycle information, including a detailed description for driving cycle information analysis, and a comprehensive summary of the latest EMMs for FCVs, with a focus on EMMs based on driving pattern recognition (DPR) and driving characteristic prediction (DCP). Based on the above analysis, an in-depth presentation of the highlights and prospects is provided for the realization of high-performance EMMs for FCVs in real-world driving situations. This paper aims at helping the relevant researchers develop suitable and efficient EMMs for FCVs using driving cycle information.

## 1. Introduction

### 1.1. Motivations

Energy shortage and environmental pollution are urgent problems that all countries in the world need to face [1]. Academic researchers and industrial engineers strive to find more green and efficient solutions for the automotive industry [2]. New energy vehicles (NEVs) are regarded as effective technologies to address the above-mentioned problem [3], and several types of NEVs have been promoted and applied, such as battery electric vehicles (BEVs) [4], plug-in hybrid electric vehicles (PHEVs) [5] and fuel cell vehicles (FCVs) [6]. In recent years, the technological progress of hydrogen energy and fuel cells (FCs) has greatly promoted the performance improvement of FCVs. FCVs have gradually become the mainstream development direction of NEVs, attracting the attention of governments and research institutes around the world [7,8]. Since FCVs typically contain two or more energy storage systems (ESSs) [9], such as a power battery pack, a fuel cell, and a super-capacitor [10], suitable and efficient energy management methods (EMMs) are critical for FCVs [11].

**Figure 1:**
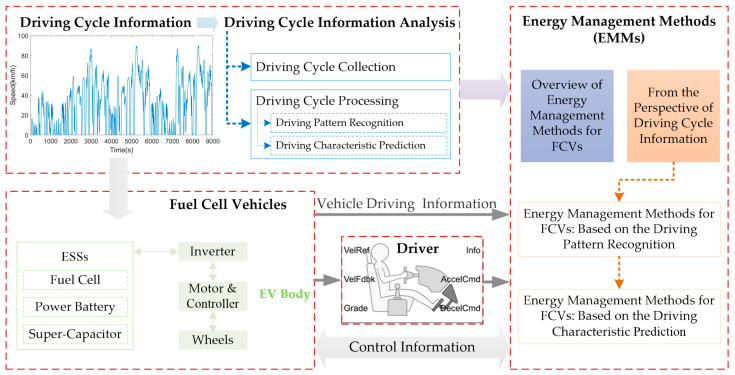
The major points of this paper.

Previous studies have shown that driving cycle information can make a difference in vehicle energy management [12,13,14]. In view of this hot topic, we have studied the characteristic relationship between energy management and the driving cycle of PHEV [15]. Driving cycle information discussed in this paper indicates the vehicle speed trajectory, which is an indication of vehicle speed versus sample time [16,17]. Driving characteristics like mileage, standard deviation of vehicle speed, acceleration, parking time ratio, driving style, and driver behaviors can be captured from driving cycle information [1,18,19]. Particularly in the energy management of FCVs, driving cycle information will directly determine the power demand, and will affect the implementation effect of energy management between the fuel cell and other ESSs [20]. Based on the above, this paper mainly focuses on the recent advances of energy management methods for fuel cell vehicles from the perspective of driving cycle information. Figure 1 depicts a summary of the major points of this paper. A total of 137 related studies were referred to in this review, and the scope, keywords and results of the literature survey in this paper are given in Table 1.

### 1.2. Contributions

Driving cycle information is very critical for the development of EMMs, mainly reflected in the current driving patterns and future driving characteristics. However, in real-world driving situations, the driving cycle changes in real time. Consequently, obtaining current and future driving cycle information is a difficult and inaccessible task. As a matter of fact, some papers have proposed some EMMs considering driving cycle information for FCVs, mainly based on driving pattern recognition and future driving characteristic prediction. It is noticed that recent reviews have stated the advances progress in energy management methods for fuel cell vehicles [21,22,23,24,25]. However, no reviews have comprehensively summarized the EMMs for FCVs from the perspective of driving cycle information.

Motivated by the literature gap, this review mainly focuses on the technologies and progress of EMMs for FCVs from the perspective of driving cycle information, and strives to be comprehensive and innovative. The main contributions of this review are as follows: (i) providing a state-of-the-art understanding of EMMs for FCVs from the perspective of driving cycle information; (ii) providing a detailed description for driving cycle information analysis, including driving cycle collection and processing; (iii) providing a comprehensive summary of the latest EMMs for FCVs, with a focus on EMMs based on driving pattern recognition and driving characteristic prediction; and (iv) providing an in-depth presentation of the important highlights and prospects regarding the innovation of EMMs for FCVs. This review hopefully accelerates the realization of high-performance EMMs for FCVs in real-world driving situations.

### 1.3. Organization

The rest of this review is organized into several sections: Section 2 mainly elaborates on driving cycle information analysis from two aspects: driving cycle collection and processing. Section 3 comprehensively summarizes energy management methods of fuel cell vehicles based on driving cycle information, with a special focus on energy management methods based on driving pattern recognition and driving characteristic prediction. Section 4 provides conclusions and prospects to accelerate the realization of high-performance energy management methods for fuel cell vehicles in real-world driving situations.

## 2. Driving Cycle Information Analysis

As the driving cycle is discerned as the input of the EMMs for FCVs, its information would affect the control performance of EMM extremely [2,26]. In addition, driving characteristics mined from driving cycle information contribute to the development and design of EMMs for FCVs. Despite having multiple typical driving cycle (such as NEDC, UDDS, EUDC, and WLTC), it is still difficult to meet the deep energy-saving needs of vehicles [27]. In order to seek larger fuel economy (FE) improvement, several studies have set out to collect and process driving cycle in the real-world driving situations [28,29,30]. 

### 2.1. Driving Cycle Collection

With the development of intelligent networking technology, information and communication technology and big data technology, it is no longer difficult to collect and analyze the driving cycle. In recent years, global positioning system (GPS) receivers [31], on board diagnostics (OBD) [32] and other onboard devices [18] have become the main devices for driving cycle collection, obtaining driving cycle information such as longitude, latitude, altitude, vehicle speed, and acceleration [33]. In addition, smartphones with built-in accelerometers, GPS receivers or any other positioning technologies are very useful for collecting driving cycle [34]. Furthermore, with the rapid development of vehicle-to-vehicle (V2V) and vehicle-to-everything (V2X) technology, an intelligent transportation system (ITS) makes the vehicle become a “mobile sensor” to collect driving cycle [35]. To be sure, driving cycle collection as the fundamental work for driving cycle analysis and processing has received much attention from some studies. A comparison of reviewed driving cycle collection studies is summarized in Table 2.

**Figure 2:**
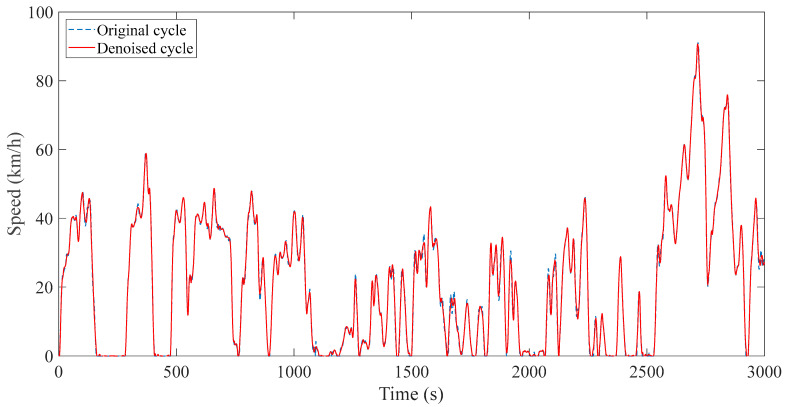
Comparison between the original and denoised data.

Due to the influence of the driving environment, jamming signals, zero drift, and buildings, the driving information collected often exhibited bad data [43]. Bad data can generally be classified into the following categories: (i) missing data, (ii) abnormal data, and (iii) burrs data [44]. In terms of the missing data and abnormal data, some studies have been conducted in detail and will not be repeated here [43,45,46]. In terms of the burrs data, the wavelet denoising method is an algorithm, which ameliorates signals with distortion, noise, and disruptions [47]. To clearly demonstrate the effectiveness of the wavelet denoising method, we selected a segment of the driving cycle and applied the wavelet denoising method, as shown in Figure 2.

### 2.2. Driving Cycle Processing

The preprocessed driving cycle can be divided into some kinematic segments to reduce the complexity of subsequent processing. This segmentation is performed for the driving cycle characteristics analysis [48]. Driving cycle characteristic parameters such as vehicle speed, maximum speed, acceleration, standard deviation of vehicle speed, and parking time ratio are often adopted to characterize driving cycle. Fewer characteristic parameters will result in the loss of necessary information, making it difficult to accurately describe driving cycle, while more characteristic parameters will increase the computational complexity. In light of this, an appropriate number of characteristic parameters should be selected. Existing studies that have found that the appropriate number of driving cycle characteristic parameters is approximately 14 include [49] (Shi et al., 12 characteristic parameters), [18] (Kayma, et al., 14 characteristic parameters), and [50] (Wang, et al., 14 characteristic parameters). However, the dimensionality of the 14 characteristic parameters is still high, and problems such as low computational efficiency and clustering difficulties exist in subsequent processing. In light of this, some studies have carried out relevant algorithms on characteristic parameters dimensionality reduction and driving cycle (kinematic segments) clustering, and the specific comparison is shown in Table 3.

#### 2.2.1. Driving Pattern Recognition

**Figure 3:**
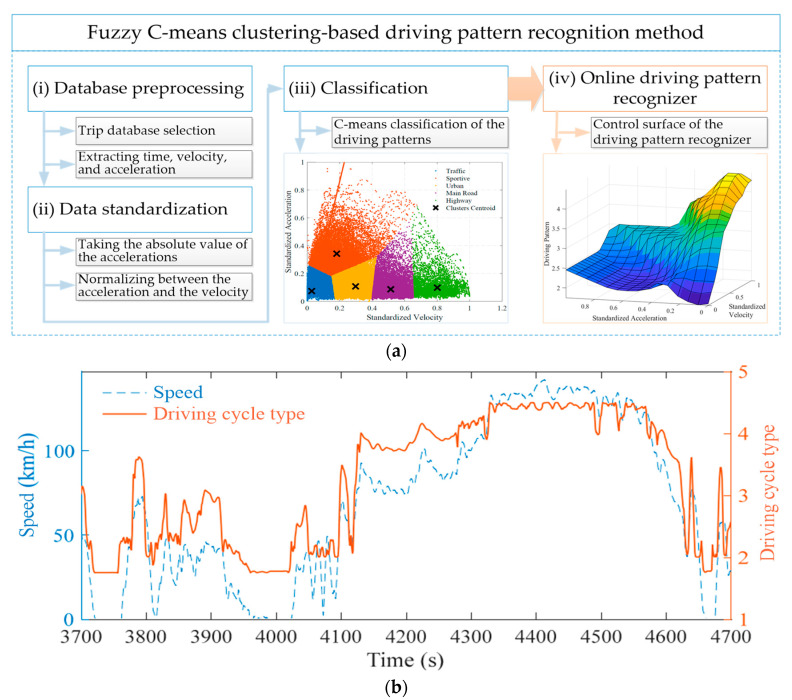
Fuzzy C-means clustering-based DPR method: (**a**) method diagram; (**b**) DPR example and result adapted from [63].

Previous studies have shown that driving pattern can greatly influence the effectiveness of EMMs [56,57,58]. For example, in the urban driving pattern, vehicles start and stop frequently, while in the highway driving pattern, vehicles often drive at a constant speed. The control parameters of one driving pattern may not be suitable for other driving patterns. In light of this, accurate and effective driving pattern recognition (DPR) can provide positive guidance for the development and design of EMMs. Some studies adopted clustering [59,60] and a fuzzy controller [61,62] to establish the driving pattern recognizer. However, the DPR effect of the clustering method is related to the initial point selection, and it is easy to fall into the local optimum. In addition, the DPR effect of the fuzzy controller relies heavily on engineering intuition, and it frequently fails to achieve satisfactory results. In [63], Matignon et al. developed the online DPR using the fuzzy C-means clustering technique. The developed driving pattern recognizer could be mainly summarized into four steps: (i) database preprocessing, (ii) data standardization, (iii) classification, and (iv) online DPR modeling, as shown in Figure 3a. The DPR method proposed in [63] adopts standardized velocity and acceleration as the input and provides the driving pattern as the output (see Figure 3b).

**Figure 4:**
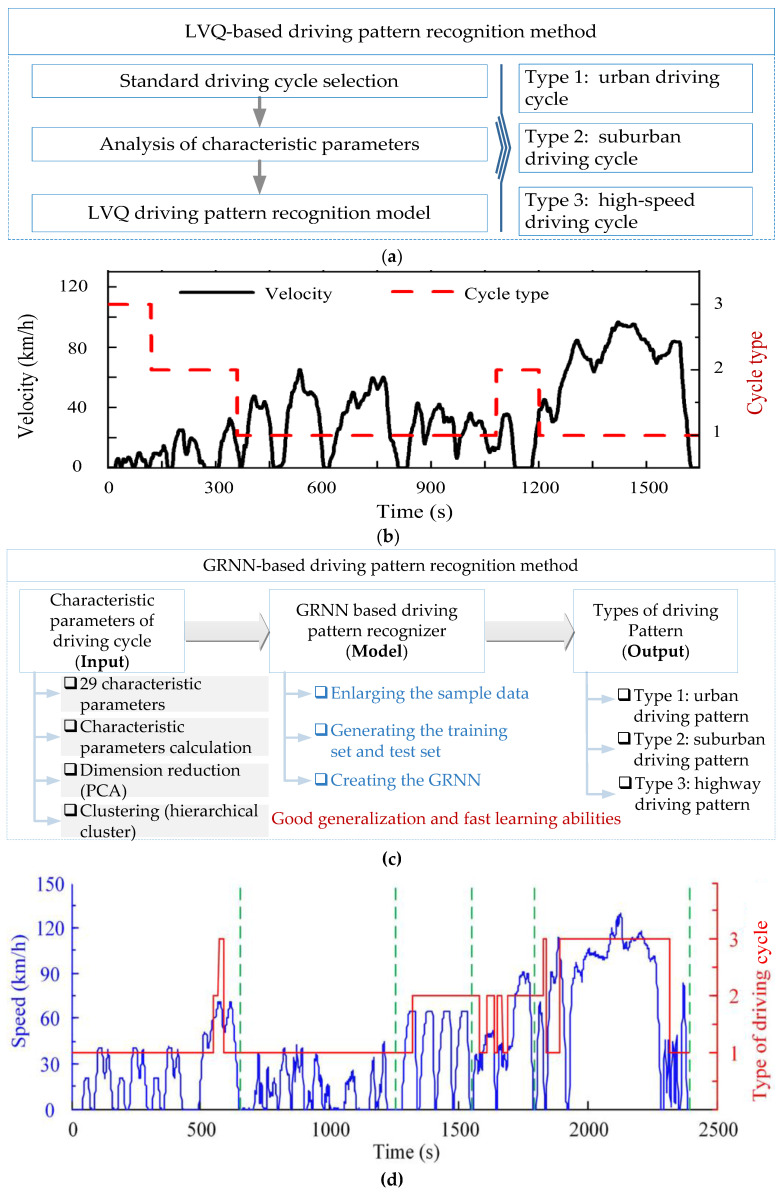
Supervised algorithms to recognize the vehicle driving pattern: (**a**) method diagram of LVQ. (**b**) DPR example and result adapted from [68]. (**c**) Method diagram of GRNN. (**d**) DPR example and result. Adapted with permission from [69]. Copyright 2019 John Wiley and Sons.

In other studies of this area, supervised algorithms are adopted to recognize the vehicle driving pattern [64], such as support vector machines (SVM) [65], learning vector quantization (LVQ) [66], and artificial neural networks (ANN) [67]. LVQ is an output forward neural network for training output, competing and output layers, which can be used for DPR. For example, Chen et al. proposed a LVQ-based driving cycle recognition method [68], and the construction process of the LVQ-based driving cycle recognizer was shown in Figure 4a. The characteristic parameters of the driving cycle were calculated and input as vectors into the LVQ-based driving cycle recognizer, and the LVQ-based driving cycle recognizer was constructed by encapsulating the LVQ neural network into a module via Simulink. The China heavy-duty commercial vehicle test cycle (CHTC) was used as an example in [68], and the driving cycle recognition results were shown in Figure 4b. In [69], a generalized regression neural network (GRNN)-based driving pattern recognizer was developed to recognize the actual driving cycle. The characteristic parameters of the driving cycle after dimensionality reduction (the PCA method) were used as inputs, and the hierarchical cluster method was adopted to obtain representative typical driving patterns. In addition, the GRNN algorithm was adopted to develop a driving pattern recognizer (see Figure 4c). Finally, the corresponding types of driving patterns are output by the model, with the example and recognition results shown in Figure 4d.

#### 2.2.2. Driving Characteristic Prediction

**Figure 5:**
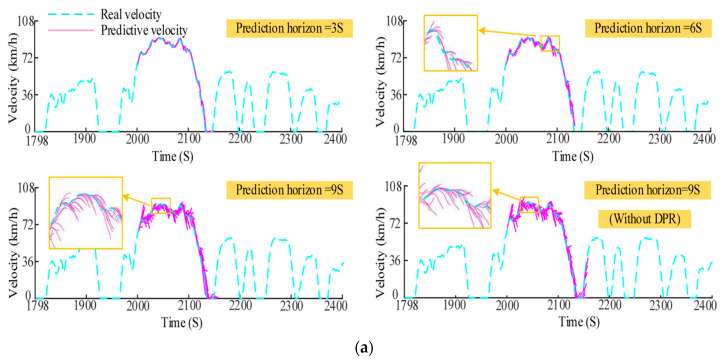

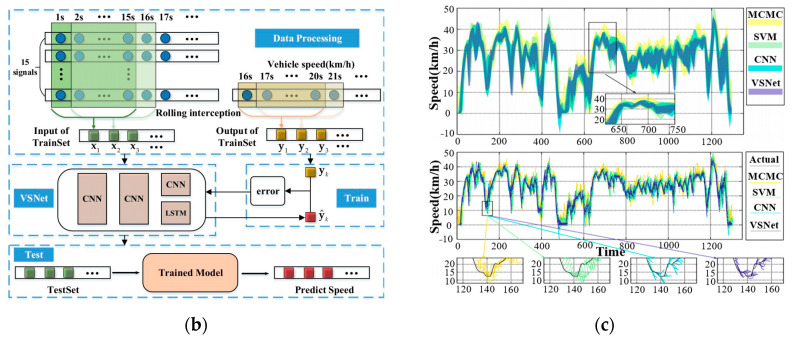
Model-based methods to predict vehicle driving characteristics: (**a**) velocity prediction results of the Markov Chain model. Adapted with permission from [76]. Copyright 2021 Elsevier. (**b**) Method diagram of VSNet. (**c**) DCP example and result of the VSNet method. Adapted from [77].

The driving cycle of a vehicle can be predicted by driving characteristic prediction (DCP) techniques, and the results indicate the current or future driving characteristics of the vehicle, like velocity and acceleration [70,71]. The effectiveness and real-time performance of DCP results have great impacts on the performance of corresponding EMMs, such as FE, lifetime of fuel cell and battery. However, DCP is a challenging study because vehicle speed is influenced by various factors, such as traffic condition and driving behavior. There are two main methods for DCP: one is model based, such as Markov Chain (MC) models [72,73] and neural network (NN) models [74,75]. Lin et al. proposed a velocity prediction method based on Markov Chain integrated with driving pattern recognition [76]. Firstly, three typical driving cycles were adopted to construct a sample driving cycle. Additionally, the K-means algorithm was adopted to cluster the constructed driving cycle segments, then the LVQ algorithm was adopted to recognize the driving pattern in real time. Finally, MC was applied to construct the Markov Transition Matrix (MTM), and the MTMs corresponding to three clustered driving patterns were adopted to predict vehicle velocity. The velocity prediction results under different prediction horizons were shown in Figure 5a, and we found that the proposed velocity prediction method was able to improve prediction accuracy effectively compared with the previous method without DPR. Xing et al. proposed a deep learning NN architecture for vehicle speed prediction, called VSNet, by combining a convolutional neural network (CNN) and a long short-term memory (LSTM) network [77]. The diagram of speed prediction was shown in Figure 5b, and the VSNet could identify the mapping relationship between vehicle signals and vehicle speed to accurately predict the future vehicle speed. The Markov Chain combined with Monte Carlo (MCMC), SVM and CNN were compared with VSNet to verify the effectiveness of VSNet in DCP, as shown in Figure 5c. The results show that VSNet outperformed the other three methods. In addition to directly using Markov or a neural network for driving characteristic prediction models, some combination methods are also proposed [78,79,80].

**Figure 6:**
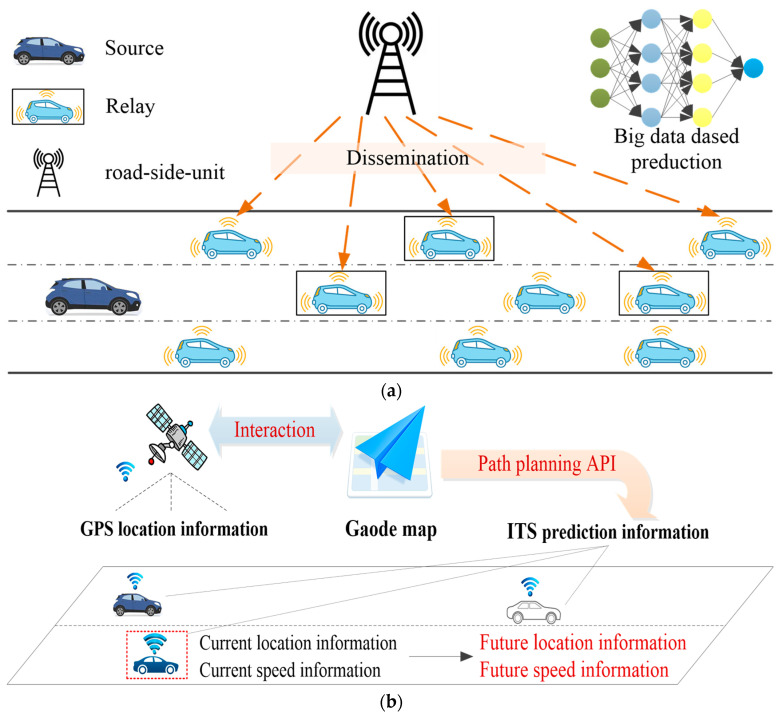
Communication technology-based methods to predict vehicle driving characteristics: (**a**) big data-assisted communication scheme. Adapted with permission from [86]. Copyright 2019 Springer Nature. (**b**) Scheme and result based on our existing work (Gaode map API).

The other is DCP based on positioning, sensing, interaction-aware, and other traffic information service technologies, such as V2X, V2V, and ITS [81,82,83]. The development of communication technologies has promoted the timely acquisition of long-term and short-term traffic information. For instance, Adelberger et al. [84] proposed a long-term velocity prediction method utilizing real-world V2X data. Hyeon et al. [85] proposed a simple and effective prediction method for generating short-term future speed trajectories using V2V information. A big data-assisted communication (BDAC) scheme for vehicular networks was proposed by An and Wu [86], and the proposed scheme is to use offline traffic data prediction to enhance the online packet forwarding procedure. The proposed scheme could be divided into two parts: the prediction part and the forwarding part, as shown in Figure 6a. The advantages of the proposed scheme in [86] over existing approaches mainly came from improving efficiency and reducing overhead. Meanwhile, it is known that accurately predicting the speed of an individual vehicle is very challenging [87]. In [87], Jiang and Fei studied the integration of traffic and vehicle driving data for individual vehicle speed prediction along specific driving routes, and proposed a novel two-level non-parametric data-driven model to improve prediction accuracy. Moreover, some studies used Baidu, Gaode, or other online map API to obtain future traffic information [88,89]. Practically, we have conducted studies on how to predict future driving characteristics based on the Gaode map API. We adopt the Gaode map API as an example [90] to construct a path-planning API call and a driving characteristic prediction scheme to obtain information such as location, mileage, and speed during future driving processes, as shown in Figure 6b.

## 3. Energy Management Methods for FCVs

As mentioned above, FCVs are one of the most promising future vehicles [91], and suitable and effective energy management methods (EMMs) are critical for energy coordination between fuel cells and power batteries/super-capacitors [92]. Therefore, the development and design of EMMs to reduce energy consumption and prolong lifespan are the subject of much research [93,94,95,96].

### 3.1. Overview of Energy Management Methods for FCVs

**Figure 7:**
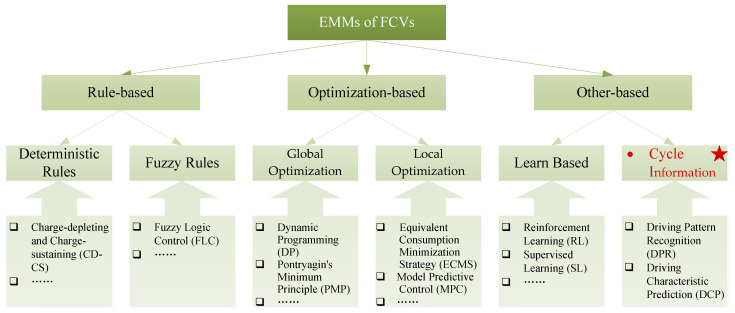
Classifications of the main EMMs for FCVs.

Previous studies have extensively studied the EMMs of FCVs to improve energy efficiency and durability. The EMMs of FCVs can be divided into three major categories: (i) rule based, (ii) optimization based, and (iii) other based, as shown in Figure 7. Rule-based EMMs are always dependent on human experiences or engineering knowledge, and they can also be divided into two categories: deterministic rules and fuzzy rules [97,98]. Although rule-based EMMs are widely adopted due to their simplicity and practicality, they cannot obtain the globally optimal solution. To achieve better management results for FCVs, numerous efforts have been made in the field of optimization-based EMMs, mainly with respect to global optimization methods and local optimization methods [99,100]. Dynamic programing (DP) and Pontryagin’s minimum principle (PMP) are classic global optimization EMMs, as they can obtain the theoretical global optimal solution [101]. However, due to non-prior driving cycle information, they cannot be directly applied to actual vehicles. To overcome these issues, the stochastic dynamic programming (SDP) and adaptive dynamic programming (ADP) methods are proposed to optimize power distribution [102,103]. Local optimization methods mainly consist of the equivalent consumption minimization strategy (ECMS) and the model predictive control (MPC) method, which can achieve real-time optimization control [97,104]. Compared to other EMMs, real-time optimization control methods are complicated, but indispensable. Similarly, to achieve near-optimal fuel economy, the adaptive equivalent consumption minimization strategy (A-ECMS) and adaptive model predictive control (A-MPC) are designed and proposed [105,106]. In addition, some papers propose combined EMMs to further improve fuel economy while ensuring FC durability, such as DP-ECMS [107], the rule-based fuzzy control method [108], adaptive neuro-fuzzy inference system-ECMS (ANFIS-ECMS) [109], and MPC-PMP [110].

As a new research hotspot in the field of artificial intelligence (AI) and internet of vehicles (IOV), learning-based and cycle information-based EMMs have been applied to achieve the optimal fuel economy of FCVs in real time [63,111]. Progress, challenges, and potential solutions of learning-based EMMs for FCVs have been reviewed in detail in [112,113,114] and will not be further elaborated here. More importantly, considering the importance of driving cycle information in the design and development of EMMs for FCVs, the following summarizes the existing papers from two perspectives: driving pattern recognition and driving characteristic prediction. To the best of our knowledge, this is the first attempt to summarize the EMMs of FCVs from the perspective of driving cycle information.

### 3.2. Energy Management Methods for FCVs: Based on Driving Pattern Recognition

**Figure 8:**
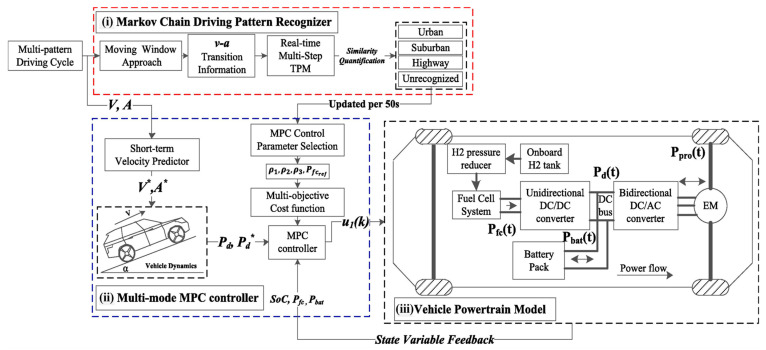
Schematic diagram of the multi-mode EMM. Adapted with permission from [119]. Copyright 2020 Elsevier.

In recent years, to improve the performance of the EMMs for FCVs, research on driving pattern recognition has been proposed [115,116,117,118,119,120]. The comparative analysis of the EMMs for FCVs based on driving pattern recognition is shown in Table 4. Particularly in [119], a multi-mode EMM for fuel cell hybrid electric vehicles was proposed. The multi-mode EMM consisted of (i) a Markov Chain driving pattern recognizer, (ii) a multi-mode MPC controller, and (iii) a vehicle powertrain model, as shown in Figure 8. In fact, each driving pattern has its own (v-a) transition characteristic. Therefore, the MC transition probability matrix (TPM) could be used to characterize the (v-a) transition behavior of each driving segment, and the MC recognizer could periodically update the pattern identification results (updated per 50 s). In addition, the offline DP algorithm was applied to carefully tune and optimize three sets of MPC control parameters. Afterwards, with the online DPR results, one set of offline-tuned MPC parameters was selected to handle the power requirement under corresponding driving patterns. The multi-mode EMM could adapt to the changeable driving conditions automatically while matching suitable MPC control parameters to achieve fuel economy and fuel cell durability improvement.

**Figure 9:**
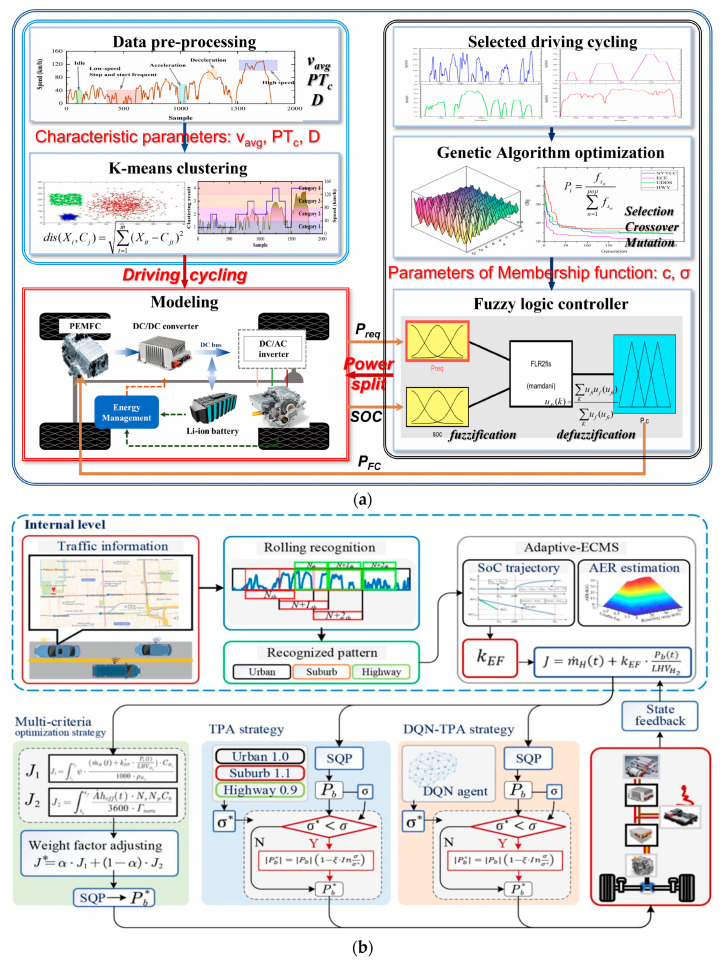
EMMs based on optimization algorithms and learning algorithms: (**a**) GA-based fuzzy optimization EMM. Adapted with permission from [121]. Copyright 2023 Elsevier. (**b**) DQN-TPA EMM. Adapted with permission from [123]. Copyright 2022 Elsevier.

Moreover, to further improve the comprehensive economy of FCVs and extend the life of the ESSs, optimization algorithms and learning algorithms were combined and adopted in the design of EMMs. In [121], a genetic algorithm (GA)-based fuzzy optimization of EMM for FCVs considering driving cycle recognition was proposed (see Figure 9a). In the proposed EMM, the K-means clustering method was developed to recognize the driving cycles, and GA was adopted to optimize the centers and widths of the fuzzy logical control membership function to overcome the limitation of the dependence on expert knowledge and improve the control efficiency of traditional fuzzy logical control. In [122], an online adaptive EMM based on DPR and regression learning was proposed. Like [121], an improved k-means cluster method was designed for DPR, which mitigated the impact of different distance definitions on clustering results. Additionally, regression learning was employed to learn the optimal control laws for DP. Finally, an online energy management regression learner chose different management models according to different patterns. To achieve equivalent hydrogen consumption minimization and battery degradation inhibition, a deep Q-learning-based trip pattern adaptive (DQN-TPA) battery longevity-conscious EMM was developed in [123]. In the proposed EMM, a learning vector quantization neural network (LVQ-NN)-based method was devised for pattern identification, and the A-ECMS was conducted to improve hydrogen consumption. Then, based on the A-ECMS, three battery longevity-conscious EMMs consisting of the multi-criteria optimization method, the TPA method, and the DQN-TPA method were developed and comprehensively discussed (see Figure 9b). Both the numerical validation and the hardware in loop (HIL) results demonstrated that the proposed DQN-TPA method could further improve hydrogen consumption and battery durability.

### 3.3. Energy Management Methods for FCVs: Based on Driving Characteristic Prediction

**Figure 10:**
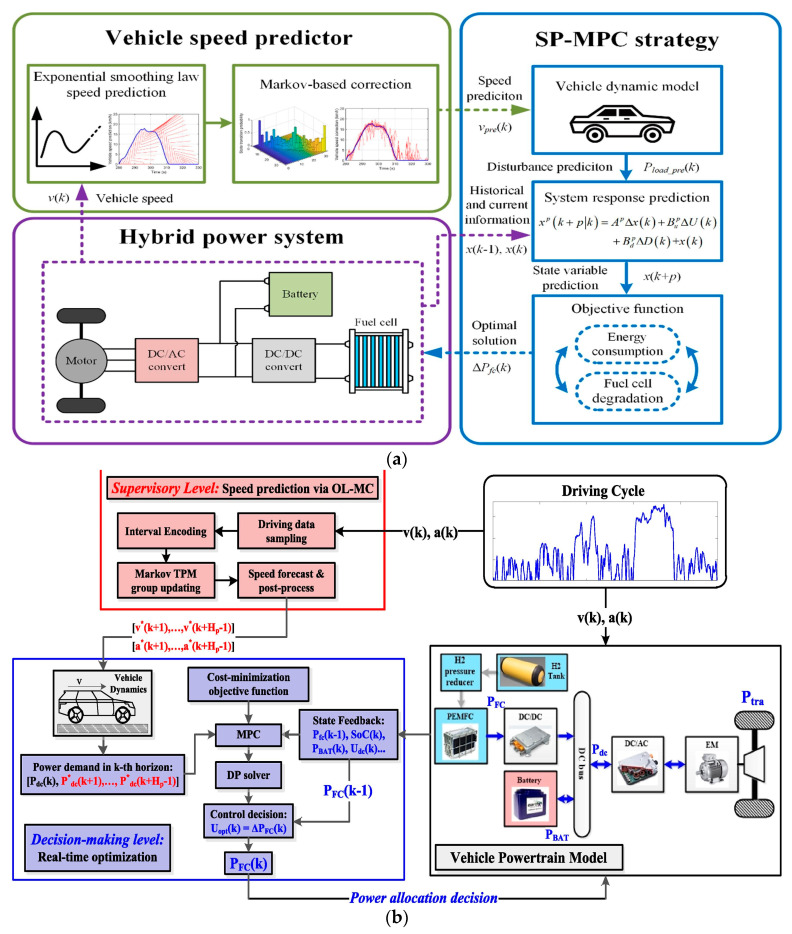
EMMs based on driving characteristic prediction: (**a**) SP-MPC EMM. Adapted with permission from [135]. Copyright 2021 Elsevier. (**b**) OL-MC MPC EMM. Adapted with permission from [137]. Copyright 2021 Elsevier.

Compared to the related research on the EMMs for FCVs based on driving pattern recognition, research on the EMMs for FCVs based on driving characteristic prediction is more extensive and in-depth due to the promotion of the intelligent process of NEVs [124,125,126,127,128,129,130,131,132]. The comparative analysis of the EMMs for FCVs based on driving characteristic prediction is shown in Table 5. The team of Sun et al. (Academician of Chinese Academy of Engineering) developed research on fuel cell system optimization control [133,134] and vehicle energy management [135,136] of FCVs based on driving speed prediction. In [135], a vehicle speed prediction model predictive control (SP-MPC) EMM was developed for FCVs (see Figure 10a). Firstly, the future vehicle total power demand was predicted through the proposed exponential smoothing law-Markov Chain vehicle speed predictor. Then, the total power demand prediction sequence was regarded as the disturbance, imported into the system response prediction model of the MPC. Finally, simulation and HIL were conducted for performance verification of the proposed SP-MPC. Correspondingly, a real-time cost-minimization strategy via speed prediction and MPC for FCVs was proposed in [137], with its schematic diagram given in Figure 10b. Firstly, upcoming vehicle speed prediction was realized by the online-learning enhanced Markov Chain (OL-MC) predictor. Then, MPC was used to quantify the vehicle’s operating cost, and DP was adopted to derive the optimal power-splitting decision over each receding horizon. Finally, the proposed method was compared against multiple benchmark methods to assess the functionality and real-time suitability.

## 4. Conclusions and Prospects

In order to improve the energy economy and prolong the powertrain system durability of FCVs, it is urgent and meaningful to develop suitable and efficient EMMs. As driving cycle information is extremely important in EMMs for FCVs, some studies have studied driving cycle information collection and processing, which lay the foundation for the development of EMMs based on driving cycle information. This paper provides a state-of-the-art understanding and a detailed overview of EMMs for FCVs from the perspective of driving cycle information. More specifically, this paper comprehensively reviews studies on driving cycle information analysis and the EMMs for FCVs, which mainly focuses on EMMs based on DPR and DCP. This paper can provide potential guidance for the design and development of EMMs for FCVs in real-world driving situations. Although great progress has been made in the EMMs based on driving cycle information for FCVs, there are still many challenges. The main prospects of this review are the following:

1. **Accurate driving pattern recognition:** The accuracy of driving pattern recognition is crucial for the development and implementation of EMMs. However, recognition accuracy and algorithm complexity are interrelated. Some advanced recognition algorithms in the existing literature have the problem of low recognition accuracy. In the future, the sampling time, the selection of characteristic parameters, and the recognition period can all be combined with advanced recognition algorithms to construct recognition methods with excellent recognition accuracy and efficiency.
2. **Short-term driving characteristic prediction:** Affected by the impacts of real-world driving conditions, the driving characteristics of vehicles will change in real time. Therefore, short-term driving characteristic prediction remains a hot and challenging issue, as it depends on various factors like the prediction method and traffic conditions. In the future, with the help of V2V, V2X, ITS and predictive algorithms, driving characteristics like speed, mileage, slope, and traffic signal light states can be predicted in the short term.
3. **Real-time energy management optimization:** Ideal energy management optimization methods can adaptively generate effective control decisions considering the DPR and DCP results. However, most current energy management optimization methods are difficult to apply to real vehicles. Advanced algorithms bring up more possibilities of real-time energy management optimization which are worth exploring. In the future, real-time/online/adaptive EMMs will be considered for supplying an excellent control effect.
4. **Integrated driving style recognition:** Even the same driver can exhibit different driving styles under different road conditions, and different driving styles can directly affect the energy management of the FCVs. Therefore, introducing the influence of driving styles into the EMMs for FCVs will be valuable and crucial. However, driving style is often described qualitatively, and is not integrated into the EMMs. In the future, integrated driving style recognition of drivers in real social driving networks will improve the effectiveness of EMMs for FCVs.

Accurate driving pattern recognition algorithms, short-term driving characteristic prediction algorithms, real-time energy management optimization methods and integrated driving style recognition methods will improve the energy economy and prolong the powertrain system durability of FCVs. In the future, our work will focus on the development and application of EMMs for FCVs based on the ITS and DCP technologies.

## Figures and Tables

**Table 1:** List of literature survey.

Scope	Keywords	Results	
Web of Science and Engineering Village (Publisher: MDPI, Elsevier, IEEE, etc.)	➢ energy management method ➢ fuel cell vehicles ➢ driving cycle information ➢ driving cycle collection ➢ driving pattern recognition ➢ driving pattern recognition	✧ website report (1)	
		✧ review papers (18)	
		✧ journal articles (118)	✓ real-world driving cycle information (60), mainly from China
			✓ non-real-world driving cycle information (58)

**Table 2:** Comparison of reviewed driving cycle collection studies.

Collection Location	Collection Device	Sampling Rate	Main Collected Information	Ref.
Chengdu, China	GPS	/	longitude, latitude, time stamp, etc.	[36]
Toronto, Canada	Qstarz BT-1000 × GPS	1 Hz	instantaneous speed, longitude, latitude, and altitude	[37]
Michigan, USA	OBD	/	latitude, longitude, vehicle speed, etc.	[38]
Islamabad, Pakistan	GPS + OBD	1 Hz	latitude, longitude, altitude, speed, road slope, etc.	[39]
Shanghai, China	Smartphones	1 Hz	altitude, average speed, average altitude, duration, etc.	[40]
Zhengzhou, China	OXTS inertial+	5 Hz	velocity, transient acceleration, and road slope	[41]
Hsinchu, China	ITS (V2V, GPS, camera, and sensors)	/	latitude, longitude, vehicle current speed, etc.	[42]

**Table 3:** Comparison of dimensionality reduction and clustering algorithms.

	Algorithm	Advantage	Disadvantage	Ref.
dimensionality reduction	principal component analysis (PCA)	simple and easy to implement, mainstream algorithm	can only extract linear characteristics, inaccurate results	[51]
	kernel principal component analysis (KPCA)	improvement of PCA, can extract non-linear characteristics	more complex and difficult to implement	[50]
	linear discriminant analysis with the diagonal eigenvalues (LDA-DE)	can efficiently handle high-dimensional data, and reduce the computation time	more complex and difficult to implement	[52]
clustering	K-means	simple and easy to implement, mainstream algorithm	slow convergence speed (non-convex dataset), not suitable for complex structure	[53]
	spectral	high computational efficiency, good convergence	selection of cluster number	[54]
	K- modified particle swarm optimization (K-MPSO)	stronger searching ability, more accurate clustering results	more complex with larger calculations	[55]

**Table 4:** Comparative analysis of the main EMMs for FCVs: based on driving pattern recognition.

EMMs	DPR Methods	Energy Sources	Simulation/ Hardware	Description	Ref.
intelligent fuzzy controller	traffic condition recognition algorithm (TCRA)	fuel cells + batteries	Advisor (UDDS/EUDC)	9~17% fuel consumption improvement vs. primary controller, and 84% correct recognition (TCRA)	[115]
adaptive fuzzy controller	neural network (NN)	fuel cells + supercapacitors	Matlab (hybrid cycles)	minimum current fluctuations and fuel consumption vs. conventional EMM, and 95% test accuracy (NN)	[116]
multi-mode EMM	LVQ neural network (NN)	fuel cells + batteries	Matlab (multi-cycle)/dynamometer testing bench	economy performance: 8.44% higher than thermostat control strategy with empirical value, 3.71% higher than thermostat control strategy optimized by the genetic algorithm (GA)	[117]
adaptive game theory controller	neural network (NN)	fuel cells + batteries + supercapacitors	Matlab (hybrid cycles)	7.4% reduction in hydrogen consumption and 23.99% reduction in battery degradation cost vs. conventional game theory controller	[118]
MPC-based multi-mode EMM	Markov Chain (MC)	fuel cells + batteries	Advisor (three multi-pattern testing cycles)	2.07~3.26% hydrogen consumption saving vs. single-mode benchmark strategy, and 94.97~98.16% identification accuracy (MC)	[119]
adaptive rule controller with optimization	vehicle operation state recognition	fuel cells + batteries + ultracapacitors	Matlab (WLTP)	33.7% increase in hydrogen consumption, 31.6% decrease in electric power consumption, and 10.94% reduction in the comprehensive operating cost vs. EMM before optimization	[120]

**Table 5:** Comparative analysis of the main EMMs for FCVs: based on driving characteristic prediction.

EMMs	DCP Methods	Energy Sources	Simulation/ Hardware	Description	Ref.
hierarchical reinforcement learning EMM	➢ long-term prediction: k-nearest neighbor (KNN) ➢ short-term prediction: feature extraction and Bayesian information criterion	fuel cells + batteries (plug-in)	Matlab (UDDS)	6.46% and 5.82% reduction in hydrogen consumption vs. CD and CS mode, respectively, and 10%~33% reduction in the fuel cell start–stop times vs. rule-based	[127]
multi-objective hierarchical prediction EMM	➢ short-term prediction: back propagation neural network (BPNN)	fuel cells + batteries (range extended)	Matlab (three testing cycles)	8.6% and 13.5% reduction in the operating costs vs. CD-CS strategy and the ECMS, respectively	[128]
integrated predictive (A-MPC) EMM	➢ short-term prediction: fuzzy C-means clustering and multi-step Markov Chain	fuel cells + batteries (range-extended plug-in)	Matlab (five testing cycles)	3.79% hydrogen consumption saving and 40.4% FC power spikes limiting vs. lower benchmark strategy, and 0.84% fuel economy deficiency and 9.18% fuel cell power transients deficiency vs. DP	[129]
real-time multi-criteria control (MPC) EMM	➢ short-term prediction: adaptive online-learning enhanced Markov	fuel cells + batteries	Matlab (multi-pattern testing cycle)	12.5% hydrogen consumption saving and 94.9% average FC power transients suppressing vs. CD-CS	[130]
sequential quadratic programming (SQP) based real-time optimization EMM	➢ short-term prediction: inflated 3D inception long short-term memory (LSTM)	fuel cells + batteries	Matlab	7.50% and 2.48% reduction in the powertrain system degradation and total cost of the energy consumption and powertrain system degradation, respectively, vs. ECMS	[131]
A-ECMS	➢ short-term prediction: long short-term memory-neural network (LSTM-NN)	fuel cells + batteries (heavy-duty vehicle)	Matlab (four driving cycles)	3.76~11.40% increase in hydrogen consumption vs. standard ECMS, but feasible for realistic conditions	[132]

## Data Availability

Not applicable.

## Abbreviations

NEV	New Energy Vehicle
BEV	Battery Electric Vehicle
PHEV	Plug-in Hybrid Electric Vehicle
FCV	Fuel Cell Vehicle
FC	Fuel Cell
ESS	Energy Storage System
EMM	Energy Management Method
NEDC	New European Driving Cycle
UDDS	Urban Dynamometer Driving Schedule
EUDC	Extra Urban Driving Cycle
WLTC	Worldwide harmonized Light-duty Test Cycle
WLTP	Worldwide harmonized Light-duty Test Procedure
CHTC	China Heavy-duty commercial vehicle Test Cycle
FE	Fuel Economy
SOC	State of Charge
OBD	On Board Diagnostics
GPS	Global Positioning System
V2V	Vehicle to Vehicle
V2X	Vehicle to Everything
ITS	Intelligent Transportation System
API	Application Programming Interface
PCA	Principal Component Analysis
KPCA	Kernel Principal Component Analysis
LDA-DE	Linear Discriminant Analysis with the Diagonal Eigenvalues
K-MPSO	K- Modified Particle Swarm Optimization
DPR	Driving Pattern Recognition
DCP	Driving Characteristic Prediction
SVM	Support Vector Machine
LVQ	Learning Vector Quantization
NN	Neural Network
ANN	Artificial Neural Network
GRNN	Generalized Regression Neural Network
BPNN	Back Propagation Neural Network
CNN	Convolutional Neural Network
LVQ-NN	Learning Vector Quantization Neural Network
LSTM	Long Short-Term Memory
LSTM-NN	Long Short-Term Memory-Neural Network
MC	Markov Chain
MTM	Markov Transition Matrix
TPM	Transition Probability Matrix
MCMC	Markov Chain combined with Monte Carlo
OL-MC	Online-Learning enhanced Markov Chain
CD-CS	Charge Depleting and Charge Sustaining
FLC	Fuzzy Logical Control
DP	Dynamic Programing
PMP	Pontryagin’s Minimum Principle
SDP	Stochastic Dynamic Programming
ADP	Adaptive Dynamic Programing
ECMS	Equivalent Consumption Minimization Strategy
MPC	Model Predictive Control
A-ECMS	Adaptive Equivalent Consumption Minimization Strategy
A-MPC	Adaptive Model Predictive Control
ANFIS-ECMS	Adaptive Neuro-Fuzzy Inference System-ECMS
RL	Reinforcement Learning
SL	Supervised Learning
AP-MPC	Speed Prediction Model Predictive Control
BDAC	Big Data-Assisted Communication
AI	Artificial Intelligence
IOV	Internet of Vehicles
TCRA	Traffic Condition Recognition Algorithm
GA	Genetic Algorithm
DQN-TPA	Deep Q-learning based Trip Pattern Adaptive
HIL	Hardware in Loop
KNN	K-Nearest Neighbor
